# Supplementary material for: Phylogenetic Investigations of Dengue 2019–2021 Outbreak in Guadeloupe and Martinique Caribbean Islands
Source: Pathogens. 2023 Sep 20;12(9):1182. doi: 10.3390/pathogens12091182 (PMC10534936; doi:10.3390/pathogens12091182)
Supplement: Supplementary file 1 [file pathogens-12-01182-s001.zip › pathogens-2531474-supplementary.pdf]

Supplementary data

Table S1. References of complete genome sequences from GenBank used in this study.

Country	Year of Collection	Serotype	Genotype	Genbank Number
Saint Martin	2021	DENV-1	V	OP895911
Florida	2020	DENV-1	V	OM909246
Ecuador	2007	DENV-1	V	OK605756
Haiti	2014	DENV-1	V	KT279761
Puerto Rico	2006	DENV-1	V	EU482591
Venezuela	1997	DENV-1	V	GU056029
Venezuela	2005	DENV-1	V	FJ810415
Argentina	2009	DENV-1	V	KC692506
Brazil	2010	DENV-1	V	JX669463
Argentina	2010	DENV-1	V	KC692516
Colombia	1999	DENV-1	V	GQ868561
Ecuador	2014	DENV-1	V	KY474307
Colombia	2008	DENV-1	V	GQ868570
Colombia	2021	DENV-1	V	OM654347
Venezuela	2015	DENV-1	V	MH450303
Peru	2021	DENV-1	V	ON123585
French Guiana	1989	DENV-1	V	AF226687
India	1963	DENV-1	V	JQ922544
India	1956	DENV-1	V	KF289073
India	2021	DENV-1	V	OQ339083
Thailand	1980	DENV-1	V	AY732476
India	1971	DENV-1	V	JQ922546
Gabon	2012	DENV-1	V	MG877557
Ivory Coast	1998	DENV-1	V	AF298807
Australia	2008	DENV-1	I	KR919821
Sri Lanka	2020	DENV-1	I	OQ080034

China	2015	DENV-1	I	MG840551
Singapore	2011	DENV-1	I	MF033196
Thailand	2017	DENV-1	I	LC410183
Cambodia	2000	DENV-1	I	GQ868637
New Caledonia	2013	DENV-1	I	MG877555
Thailand	1986	DENV-1	I	JN638336
Vietnam	2008	DENV-1	I	FJ410214
Taiwan	1994	DENV-1	I	AB608788
Malaysia	2005	DENV-1	I	JN697057
Thailand	1960	DENV-1	II	JQ922547
Thailand	1964	DENV-1	II	AF180817
Malaysia	1972	DENV-1	III	EF457905
Philippines	2016	DENV-1	IV	LC128301
Nauru Island	1974	DENV-1	IV	U88535
Micronesia	2004	DENV-1	IV	AB204803
Indonesia	1998	DENV-1	IV	AB189121
Seychelles	2003	DENV-1	IV	AB195673
Reunion island	2004	DENV-1	IV	DQ285560
French Polynesia	2008	DENV-1	IV	MG181997
Hawai	2001	DENV-1	IV	DQ672562
Malaysia	2005	DENV-1	IV	JN697056
Easter Island	2002	DENV-1	IV	EU863650
Reunion Island	2018	DENV-2	Cosmopolitan	MN272404
India	2016	DENV-2	Cosmopolitan	MK858111
Singapore	2017	DENV-2	Cosmopolitan	MW512468
China	2017	DENV-2	Cosmopolitan	MH827529
Saudi Arabia	2016	DENV-2	Cosmopolitan	MN294937
India	2001	DENV-2	Cosmopolitan	DQ448231
Pakistan	2009	DENV-2	Cosmopolitan	KF041237
Seychelles	2016	DENV-2	Cosmopolitan	MN272405

Australia	1993	DENV-2	Cosmopolitan	AY037116
Burkina Faso	2016	DENV-2	Cosmopolitan	KY627763
China	2003	DENV-2	Cosmopolitan	FJ196853
Indonesia	1975	DENV-2	Cosmopolitan	GQ398258
Indonesia	1998	DENV-2	Cosmopolitan	AB189124
Brunei	2005	DENV-2	Cosmopolitan	EU179857
Singapore	2016	DENV-2	Cosmopolitan	MW512449
India	2013	DENV-2	Cosmopolitan	MH822956
China	2018	DENV-2	Cosmopolitan	MK783206
France	2014	DENV-2	Cosmopolitan	MN720746
India	2016	DENV-2	Cosmopolitan	MK858107
India	2016	DENV-2	Cosmopolitan	OQ339137
India	2019	DENV-2	Cosmopolitan	OP310805
Malaysia	1970	DENV-2	Sylvatic	EF105379
Burkina Faso	1980	DENV-2	Sylvatic	EF105386
French Guiana	1993	DENV-2	Asian/American	EU920828
Venezuela	2015	DENV-2	Asian/American	MH069495
Colombia	2019	DENV-2	Asian/American	MZ773408
Venezuela	2007	DENV-2	Asian/American	HQ332190
Colombia	2021	DENV-2	Asian/American	ON510270
Nicaragua	2001	DENV-2	Asian/American	GQ199898
Cuba	1997	DENV-2	Asian/American	AY702037
Thailand	1995	DENV-2	Asian I	GQ868543
Vietnam	2003	DENV-2	Asian I	EU482782
Thailand	2001	DENV-2	Asian I	FJ744714
Cambodia	2005	DENV-2	Asian I	FJ639708
Vietnam	2004	DENV-2	Asian I	FM210231
Vietnam	2004	DENV-2	Asian I	EU482774
China	2015	DENV-2	Asian I	KY672945
Laos	2010	DENV-2	Asian I	KY849763

Thailand	2001	DENV-2	Asian I	DQ181797
Thailand	2016	DENV-2	Asian I	LC410184
Thailand	1964	DENV-2	Asian I	GQ868591
China	2017	DENV-2	Asian II	OM256481
Indonesia	1975	DENV-2	Asian II	GQ398268
Papua New Guinea	1944	DENV-2	Asian II	KM204118
Taiwan	2008	DENV-2	Asian II	HQ891024
Phillipines	1988	DENV-2	Asian II	OK605760
Palaos	1988	DENV-2	Asian II	OK469347
Mexico	1983	DENV-2	American	GQ868589
Colombia	1986	DENV-2	American	GQ868592
Colombia	1986	DENV-2	American	AY702040
Fiji	1971	DENV-2	American	HM582099
French Polynesia	1972	DENV-2	American	HM582108
American Samoa	1972	DENV-2	American	HM582104
Tonga	1974	DENV-2	American	HM582112
Venezuela	1987	DENV-2	American	GQ868599
Puerto Rico	1969	DENV-2	American	JX966380
New Caledonia	1972	DENV-2	American	HM582103
China	2016	DENV-3	III	MN018385
India	2016	DENV-3	III	MK858153
India	2019	DENV-3	III	ON123658
Singapore	2013	DENV-3	III	OP410994
Singapore	2019	DENV-3	III	OP410997
Pakistan	2006	DENV-3	III	KF041259
Thailand	2016	DENV-3	III	LC410192
India	2008	DENV-3	III	GQ466079
China	2009	DENV-3	III	JN662391
Brazil	2006	DENV-3	III	OQ727062
Brazil	2023	DENV-3	III	OQ706226

India	2022	DENV-3	III	OQ721955
Ethiopia	2019	DENV-3	III	ON890788
Senegal	2009	DENV-3	III	KU509282
Burkina Faso	2017	DENV-3	III	MT261978
Gabon	2016	DENV-3	III	LC379196
India	2021	DENV-3	III	ON109599
Bhutan	2019	DENV-3	III	OM865816
Thailand	2017	DENV-3	III	LC410195
China	2019	DENV-3	III	MW720883
Maldives	2019	DENV-3	III	ON890789
Singapore	2012	DENV-3	III	KX380841
French Polynesia	1989	DENV-3	I	AY744677
East Timor	2005	DENV-3	I	AB214882
Indonesia	2008	DENV-3	I	KC762689
Indonesia	2016	DENV-3	I	MH823209
French Polynesia	1992	DENV-3	I	AY744683
Cook Islands	1991	DENV-3	I	FJ898455
Samoa	1995	DENV-3	I	FJ898456
Indonesia	2007	DENV-3	I	KC762684
Papua New Guinea	2008	DENV-3	I	KY794789
Indonesia	1998	DENV-3	II	DQ675520
Thailand	1993	DENV-3	II	AY676351
Thailand	2012	DENV-3	II	MH888332
Bangladesh	2002	DENV-3	II	AY496873
Thailand	1998	DENV-3	II	AY676348
Thailand	1994	DENV-3	II	AY923865
Taiwan	1998	DENV-3	II	KY670634
Puerto Rico	1963	DENV-3	IV	MW945427
China	2000	DENV-3	V	AF317645
Philippines	1956	DENV-3	V	KU050695

Philippines	1964	DENV-3	V	KM190937
United States	1963	DENV-3	V	JQ922554
Brazil	2006	DENV-3	V	JN697379
Brazil	2007	DENV-3	V	EF629370
